# A Lysine Residue at the C-Terminus of MHC Class I Ligands Correlates with Low C-Terminal Proteasomal Cleavage Probability

**DOI:** 10.3390/biom13091300

**Published:** 2023-08-24

**Authors:** Adrian Schmalen, Ilona E. Kammerl, Silke Meiners, Elfriede Noessner, Cornelia A. Deeg, Stefanie M. Hauck

**Affiliations:** 1 Chair of Physiology, Department of Veterinary Sciences, LMU Munich, Martinsried, 82152 Planegg, Germany; 2 Core Facility—Metabolomics and Proteomics Core, Helmholtz Center Munich, German Research Center for Environmental Health (GmbH), 80939 Munich, Germany; 3 Comprehensive Pneumology Center (CPC), University Hospital, Ludwig-Maximilians-University, Helmholtz Center Munich, Member of the German Center for Lung Research (DZL), 81377 Munich, Germany; 4 Research Center Borstel, Leibniz Lung Center, Airway Research Center North (ARCN), Member of the German Center for Lung Research (DZL), 23845 Borstel, Germany; 5 Institute of Experimental Medicine, Christian-Albrechts University Kiel, 24118 Kiel, Germany; 6 Immunoanalytics Research Group—Tissue Control of Immunocytes, Helmholtz Center Munich, 81377 Munich, Germany

**Keywords:** immunopeptidomics, proteasome independent, non-canonical MHC class I ligands, HLA-A*03:01, HLA-A*11:01, HLA-A*30:01, proteasomal cleavage probability, peptide origin, peptide processing

## Abstract

The majority of peptides presented by MHC class I result from proteasomal protein turnover. The specialized immunoproteasome, which is induced during inflammation, plays a major role in antigenic peptide generation. However, other cellular proteases can, either alone or together with the proteasome, contribute peptides to MHC class I loading non-canonically. We used an immunopeptidomics workflow combined with prediction software for proteasomal cleavage probabilities to analyze how inflammatory conditions affect the proteasomal processing of immune epitopes presented by A549 cells. The treatment of A549 cells with IFNγ enhanced the proteasomal cleavage probability of MHC class I ligands for both the constitutive proteasome and the immunoproteasome. Furthermore, IFNγ alters the contribution of the different HLA allotypes to the immunopeptidome. When we calculated the HLA allotype-specific proteasomal cleavage probabilities for MHC class I ligands, the peptides presented by HLA-A*30:01 showed characteristics hinting at a reduced C-terminal proteasomal cleavage probability independently of the type of proteasome. This was confirmed by HLA-A*30:01 ligands from the immune epitope database, which also showed this effect. Furthermore, two additional HLA allotypes, namely, HLA-A*03:01 and HLA-A*11:01, presented peptides with a markedly reduced C-terminal proteasomal cleavage probability. The peptides eluted from all three HLA allotypes shared a peptide binding motif with a C-terminal lysine residue, suggesting that this lysine residue impairs proteasome-dependent HLA ligand production and might, in turn, favor peptide generation by other cellular proteases.

## 1. Introduction

The turnover of aged or misfolded proteins or the degradation of defective ribosomal products results in a pool of peptides that serve as substrates for major histocompatibility complex (MHC) class I [1,2,3]. MHC class I consists of a membrane-bound α chain encoded by one of three canonical class I human leucocyte antigen (HLA) genes, the β2-microglobulin and the aforementioned peptide [4,5,6]. The presentation of the peptides at the cell surface allows CD8^+^ T cells to inspect for “non-self” peptides that might originate from intracellular pathogens or transformed cells [7]. Peptide generation for antigen presentation by MHC class I is canonically performed by the proteasome [1]. The proteasome has three distinct catalytic cleavage activities and a broad substrate specificity. Based on structural similarities and their cleavage site specificity, catalytically active sites are designated as chymotrypsin-like, trypsin-like and caspase-like activities, respectively [8,9,10]. However, other cellular proteases, like nardilysin [11], tripeptidyl-peptidase II [12] or the insulin-degrading enzyme [13], can complement the peptide processing of the proteasome. Additionally, several types of proteasomes are described, namely, the constitutive proteasome (c20S), the immunoproteasome (i20S) and the thymoproteasome (t20S) [14,15,16]. While c20S is ubiquitously expressed, the i20S is constitutively expressed in immune cells and in cells stimulated with cytokines like Interferon γ (IFNγ) [14,15,17]. The expression of the t20S is limited to the thymus [16]. Furthermore, mixed or intermediate types of proteasomes are described, where catalytically active subunits of the c20S combine with catalytically active subunits of the i20S [18]. Besides the degradation of proteins, the proteasome can splice peptides, further enlarging the already complex peptide composition of the immunopeptidome [19].

Cytosolic peptides are actively transported into the lumen of the endoplasmatic reticulum (ER) via the Transporter Associated with Antigen Processing (TAP) [20]. The TAP itself is part of the MHC class I peptide loading complex that mediates the proper loading of peptides into the peptide binding cleft of MHC class I [21,22]. The peptides presented by MHC class I are between 8 and 14 amino acids in length. Although the proteasome can produce peptides that suit the length requirements of MHC class I, most peptides undergo further processing within the ER by N-terminal aminopeptidases like the ER aminopeptidase associated with antigen processing (ERAP1) or ER aminopeptidase 2 (ERAP2) [23,24]. Because there are no C-terminal aminopeptidases, the C-terminus of peptides is not processed further, while the N-terminus can be trimmed during peptide loading. Consequently, the C-terminus is predominantly of proteasomal origin, while the N-terminus is not [25,26].

Like other omics approaches that aim to describe the steady state of a given system and the relationship of its components, immunopeptidomics does so for the immune epitopes presented by a cell [27]. To access the immunopeptidome of a cell, MHC class I is immunoprecipitated after cell lysis, followed by peptide elution and purification from the precipitated complexes. Subsequently, MHC class I peptides are identified via mass spectrometry [27,28]. Due to the high complexity of the immunopeptidome and the limited amount of MHC class I per cell, analyses of the immunopeptidome require a large amount of input material. Consequently, several approaches are undertaken to allow for the prediction of the immunopeptidome in silico to overcome the costs and time demands of immunopeptidomics. One such tool is the open-source software Pepsickle (version 0.1.2), which allows one to calculate the cleavage probability of proteins by the c20S or the i20S [29]. Pepsickle utilizes a deep ensemble learning algorithm together with a set of experimental proteasomal training data. Furthermore, the tool considers the upstream and downstream amino acid context at each cleavage site for its calculation. Pepsickle allows for a fast and reliable calculation of the estimated peptide pool generated by the proteasome that will predominantly be loaded onto MHC class I [29].

While Pepsickle was originally intended to predict the peptides presented by MHC class I, we used Pepsickle here to estimate the origin of each peptide of the immunopeptidome. In the process, we identified three HLA allotypes that presented peptides with a drastically diminished C-terminal proteasomal cleavage probability. Besides their reduced C-terminal proteasomal cleavage probability, these peptides shared a C-terminal lysine residue (K) as a common feature. We therefore speculate that the HLA allotypes HLA-A*03:01, HLA-A*11:01 and HLA-A*30:01 have an increased likelihood of presenting peptides that do not originate solely from proteasomal degradation.

## 2. Materials and Methods

### 2.1. Cell Line

The human lung epithelial-like adenocarcinoma cell line A549 was cultured with DMEM supplemented with 10% fetal bovine serum (Biochrom/Merck, Taufkirchen, Germany) and 1 × Pen/Strep (Gibco/Thermo Fisher Scientific, Dreieich, Germany) in 15 cm dishes in a humidified atmosphere with 5% CO_2_ at 37 °C. Eleven dishes per replicate were used for control and IFNγ treatment. On the third day after seeding, the cells were treated with 75 IU/mL recombinant human IFNγ (Merck, Taufkirchen, Germany) for 24 h. The cells of 10 dishes per treatment were then scraped in PBS and centrifuged, and pellets were stored at −80 °C for analysis. The cells of one dish per treatment were trypsinized and counted for the estimation of the number of cells per dish.

### 2.2. MHC Class I Immunoprecipitation and Peptide Elution

One hundred million cells per sample were lysed with a buffer containing 0.25% (*w*/*v*) sodium deoxycholate, 0.2 mM iodoacetamide, 1 mM EDTA, 1 × cOmplete Protease Inhibitor Cocktail (Merck, Taufkirchen, Germany), 1 mM Phenylmethyl-sulfonylfluoride and 1% Octyl-beta-D-glucopyranoside in PBS [28]. Then, 10 µg lysate per sample was used for a parallel proteome analysis. 

The lysates for the immunopeptidomics analysis were cleared via centrifugation (51,200× *g* for 50 min at 4 °C), and native MHC class I complexes were precipitated with the pan-MHC class I-specific antibody W6/32 (HB-95, ATCC/ LGC Standards, Wesel, Germany) coupled to protein G sepharose 4 Fast Flow beads (Merck, Taufkirchen, Germany) as described [28]. Co-precipitated immunopeptides were eluted with 1% (*v*/*v*) trifluoroacetic acid (TFA) and purified using Sep-Pak tC18 columns containing 100 mg sorbent (Waters, Eschborn, Germany). The elution of peptides from the tC18 sorbent was conducted in two steps: first with 28% acetonitrile (ACN) in 0.1% TFA and consecutively with 32% ACN in 0.1% TFA. Both eluates were volume-reduced using a vacuum evaporator until almost all liquid was evaporated. The peptides were then resolved with 2% ACN in 0.5% TFA and stored at −80 °C until further analysis [28].

### 2.3. LC-MS/MS and Quantitative Analysis

An LC-MS/MS analysis was performed on a QExactive HF-X mass spectrometer (Thermo Fisher Scientific, Dreieich, Germany) online coupled to an UItimate 3000 RSLC nano-HPLC (Dionex/Thermo Fisher Scientific, Dreieich, Germany). The peptides were automatically injected and loaded onto a C18 trap column (300 µm inner diameter (ID) × 5 mm, Acclaim PepMap100 C18, 5 µm, 100 Å, LC Packings; Thermo Fisher Scientific) at a 30 µL/min flow rate prior to performing C18 reversed-phase chromatography on the analytical column (nanoEase MZ HSS T3 Column, 100 Å, 1.8 µm, 75 µm × 250 mm, Waters, Eschborn, Germany) at a 250 nL/min flow rate in a 95 min non-linear acetonitrile gradient from 3 to 40% in 0.1% formic acid. Profile precursor spectra from 300 to 1650 *m*/*z* were recorded at 60,000 resolution with an automatic gain control (AGC) target of 3 × 10^6^ and a maximum injection time of 30 ms. The 15 most abundant peptide ions of charges 1 to 5 were selected from the MS scan and fragmented using HCD with a normalized collision energy of 28, an isolation window of 1.6 *m*/*z* and a dynamic exclusion of 15 s. MS/MS spectra were recorded at a resolution of 30,000 with an AGC target of 1 × 10^5^ and a maximum injection time of 50 ms. This method was adapted for proteome measurements as follows: Peptides from the FASP digestion of 10 µg lysate were automatically injected and loaded onto a C18 trap column (300 μm inner diameter (ID) × 5 mm, Acclaim PepMap100 C18, 5 μm, 100 Å, LC Packings) at a 30 μL/min flow rate prior to performing C18 reversed-phase chromatography on the analytical column (nanoEase MZ HSS T3 Column, 100 Å, 1.8 μm, 75 μm × 250 mm, Waters) at a 250 nL/min flow rate in a 95 min non-linear acetonitrile gradient from 3 to 40% in 0.1% formic acid. By recording the profile precursor spectrum from 300 to 1500 *m*/*z* at 60,000 resolution, the top 15 fragment spectra of charges 2 to 7 were recorded at 15 000 resolution and a dynamic exclusion for 30 s [30].

Proteome Discoverer 2.5 software (version 2.5.0.400; Thermo Fisher Scientific, Dreieich, Germany) was used for peptide and protein identification via a database search (Sequest HT search engine) against the SwissProt human database (Release 2020_02, 20,435 sequences; 11,490,581 residues). Furthermore, the workflow for the identification of the immunopeptidome included the INFERYS rescoring node [31]. The database search was performed with an unspecified peptide cleavage. The precursor mass tolerance was 10 ppm, and the fragment mass tolerance was 0.02 Da. The carbamidomethylation of cysteine was set as static modification. Dynamic modifications included the deamidation of asparagine and glutamine, the oxidation of methionine (M) and a combination of M loss with acetylation on the protein N-terminus. Peptide spectrum matches and peptides were validated with the Percolator algorithm [32].

The immunopeptidomes of three independent experiments measured in technical duplicates were analyzed. Only the top-scoring hits for each spectrum were accepted with a false discovery rate (FDR) < 1% (high confidence). 

In total, we identified 2530 peptides with an MHC class I binding rank ≤ 2% presented by the A549 cells. Furthermore, 1625 MHC class I ligands were frequently detected, meaning that they were identified in at least three out of six samples with a high confidence peptide spectrum match or in more than 50% of the samples with lower confident peptide spectrum matches.

The mass spectrometry raw files used in this study were originally produced for a previous publication, where they were utilized to address the influence of cigarette smoke on the antiviral T-cell immune response [30]. Here, we reanalyzed the data to investigate the processing of MHC class I ligands by the c20S or the i20S.

### 2.4. Cleavage Probability Prediction

The MHC class I binding ranks for each peptide were calculated using the MixMHCpred tool (version 2.1; Gfeller lab, Lausanne, Switzerland; available online at no charge from https://github.com/GfellerLab/MixMHCpred, accessed on 22 August 2023) [33,34]. MixMHCpred calculates the peptides’ binding scores for a previously defined set of HLA allotypes. The binding rank indicates the likelihood that a random set of peptides would have a similar or better binding score for a given HLA allotype [33,34]. The peptides were defined as HLA-specific binders when they were between 8 and 14 amino acids in length and had a binding rank of less than or equal to 2%. Gibbs clustering was performed with the Linux-based Gibbs Clustering tool (version 2.0f; Technical University of Denmark, Denmark; available online at no charge from https://services.healthtech.dtu.dk/service.php?GibbsCluster-2.0, accessed on 22 August 2023), and the recommended settings for MHC class I peptides were used [35,36]. Sequence logos were visualized using the R package ggseqlogo (version 0.1) [37]. 

The proteasomal cleavage probability for processing by the c20S and the i20S for all proteins contained in the SwissProt human database (Release 2020_02, 20,435 sequences; 11,490,581 residues) was determined based on complete protein sequences using the software Pepsickle [29]. Pepsickle provides the cleavage probabilities for all amino acids of a given protein sequence, taking into account the upstream and downstream amino acid context. Thus, we generated a database containing the proteasomal cleavage probabilities for all amino acid positions of the SwissProt human database. The C-terminal and N-terminal c20S and i20S cleavage probabilities of the detected HLA-specific immune epitopes (MHC class I binding rank ≤ 2%) were retrieved from this newly generated database.

Furthermore, we retrieved 554,617 MHC class I ligands from the Immune Epitope Database (IEDB) as of 8 March 2022 (available online at https://www.iedb.org/, accessed on 8 March 2022) [38]. We were able to calculate the C-terminal and N-terminal c20S and i20S proteasomal cleavage probabilities for 88,513 MHC peptides from the IEDB assigned to 71 HLA allotypes.

### 2.5. Statistical Analysis

Data are shown in the figures as mean ± standard deviation (SD), and the comparison between two groups was analyzed as detailed in the figure legends, with statistical significance indicated as * *p* < 0.05, ** *p* < 0.01, *** and *p* < 0.001. A statistical evaluation of the immunopeptidome analysis was performed using R studio 2021.09.1 and R version 4.1.2 (1 November 2021). Non-parametric aligned rank transform ANOVA was performed using the R package ARTool (version 0.11.1), followed by the aligned rank transform contrasts post hoc test and adjustment for multiple testing according to Benjamini and Hochberg [39]. The non-parametric Mood’s median test followed by Mood’s pairwise median test was performed using the R package RVAideMemoire (version 0.9-81-2). Non-parametric Wilcoxon/Mann–Whitney U test was performed using the R package Rstatix (version 0.7.0). The *p* values were adjusted for multiple testing according to Benjamini and Hochberg [39], unless otherwise specified.

## 3. Results

IFNγ induces the expression of the i20S affecting the proteasomal cleavage preferences and, thus, shapes the peptide pool available for presentation by MHC class I [40]. Thus, we planned to investigate the direct influence of different types of proteasomes on individual peptides of the immunopeptidome. Therefore, we isolated and identified the immunopeptidome of A549 cells treated with 75 IU/mL IFNγ for 24 h, which induces the immunoproteasome (Supplementary Table S1) [41]. Untreated cells served as a control. 

Furthermore, 10 µg of each lysate was used for a proteome analysis in parallel to the immunopeptidome analysis. Thus, we were able to investigate the influence of the IFNγ treatment on the proteins expressed by the A549 cells. We identified in total 5477 proteins in the proteome of the A549 cells treated with or without IFNγ (Supplementary Table S2). In total, 238 proteins were differentially expressed in the A549 cells treated with IFNγ, with a log2 abundance ratio greater or equal to 1 or less than or equal to −1 and an adjusted *p*-value smaller than 0.05, as shown previously (Supplementary Figure S1) [30]. Amongst the differentially expressed proteins were components of the i20S and of the MHC class I antigen presentation machinery (Table 1).

**Figure 1:**
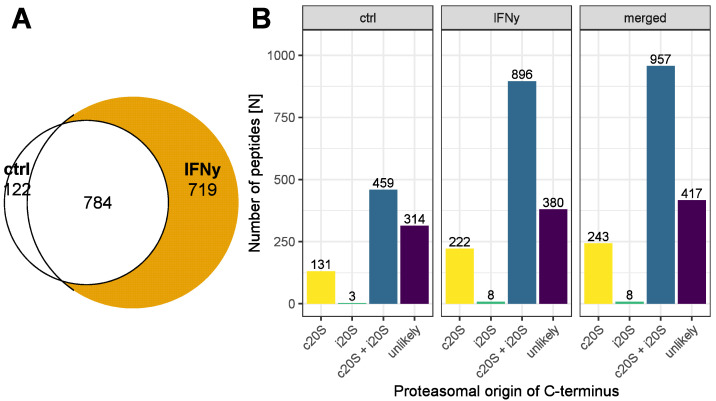
Composition of the HLA class I ligands derived from A549 cells regarding the proteasomal origin of their C-termini**.** Venn diagram of frequently identified MHC class I ligands derived from untreated A549 cells (ctrl; blue) and A549 cells treated with 75 IU/mL IFNγ for 24 h (IFNγ, yellow) (**A**). The C-terminal proteasomal cleavage probabilities for processing of the MHC class I ligands by the c20S and i20S were determined using the software Pepsickle [29]. Peptides were categorized as c20S-exclusive (yellow), i20S-exclusive (green), processed by either c20S or i20S (c20S + i20S; blue), or having a proteasomal cleavage probability smaller than 0.5 (unlikely; purple). Bar plot of the number of peptides in each category isolated from untreated cells (ctrl), IFNγ-treated cells (IFNγ) or all frequently identified HLA class I ligands (merged) (**B**).

The MHC class I binding ranks for the identified peptides of the immunopeptidome of the A549 cells treated with IFNγ or untreated controls were calculated with the software MixMHCpred, and the peptides with a binding rank < 2% were accepted as MHC class I-specific immune epitopes [33,34]. In total, we detected 1625 immune epitopes in at least 50% of the analyzed samples. Furthermore, 1503 of those peptides were presented by the IFNγ-treated cells, and 906 were presented by the untreated control, with an overlap of 784 immune epitopes presented by both cells (Figure 1A).

The software Pepsickle [29] was used to calculate the C- and N-terminal cleavage probabilities of these peptides by the c20S and by the i20S. In total, 243 frequently identified immune epitopes had a C-terminal cleavage probability suggesting processing by the c20S but not by the i20S, and 8 frequently detected peptides suggested processing by the i20S but not by the c20S. Furthermore, 417 peptides had a cleavage probability smaller than 0.5, which is the default threshold of the software Pepsickle for proteasomal processing [29]. The treatment of the A549 cells with IFNγ increased the antigen presentation of peptides with a C-terminal proteasomal cleavage probability suggesting processing exclusively by the c20S, exclusively by the i20S or by both types of proteasomes. Furthermore, the IFNy treatment slightly increased the antigen presentation of peptides with a C-terminal cleavage probability smaller than 0.5 for both types of proteasomes (Figure 1B).

**Figure 2:**
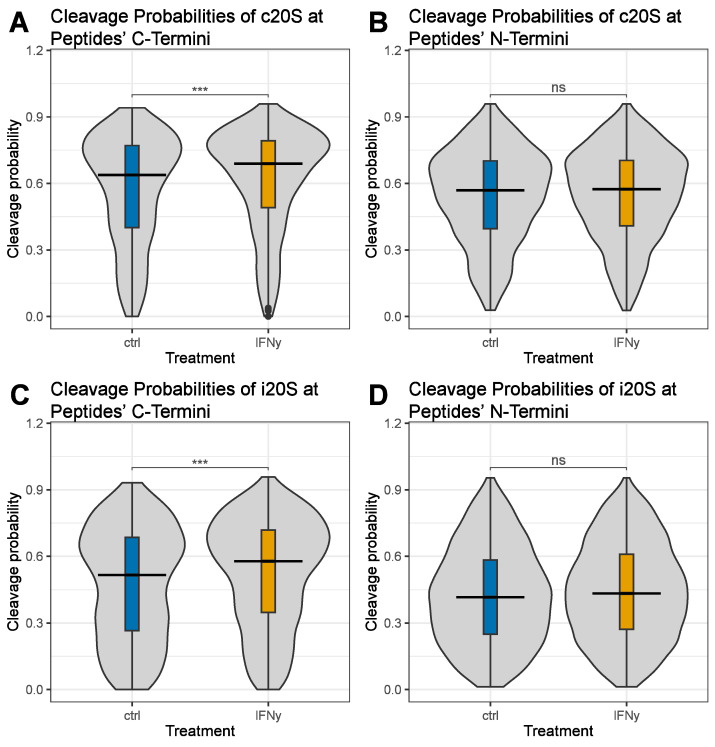
C- and N-terminal cleavage probabilities of MHC class I-presented peptides derived from A549 cells. A549 cells were treated with 75 IU/mL IFNγ (yellow). Untreated cells served as a control (ctrl; blue). MHC class I-presented peptides were isolated and identified using mass spectrometry. Cleavage probabilities for frequently identified peptides with an MHC class I binding rank ≤ 2% were calculated using the software Pepsickle. Depicted are trimmed violin plots containing a boxplot of the C- and N-terminal cleavage probabilities for the c20S (**A**,**B**) and i20S (**C**,**D**). The boxplots show median, first and third quartiles, and whiskers indicating data within the ±1.5 times interquartile range. Non-parametric Wilcoxon/Mann–Whitney U tests were performed to test for statistical significance, and *p*-values were adjusted for multiple testing according to Bonferroni, *** *p* < 0.001, ns *p* ≥ 0.05.

The N-terminus of immunogenic peptides is trimmed during peptide loading by N-terminal aminopeptidases like ERAP1 and is typically not generated by the proteasome [42]. In contrast, there are no C-terminal aminopeptidases, and the majority of the C-termini of MHC class I ligands are of proteasomal origin [26]. In line with this, the median proteasomal cleavage probabilities of the MHC class I peptides of both types of proteasomes were higher for the C-terminal cleavage sites than for the N-terminal cleavage sites (Figure 2). Depicted in Figure 2 are violin plots of the C-terminal c20S cleavage probabilities (Figure 2A), the N-terminal c20S cleavage probabilities (Figure 2B), the C-terminal i20S cleavage probabilities (Figure 2C) and the N-terminal i20S cleavage probabilities (Figure 2D) of the frequently identified immune epitopes presented by the A549 cells treated with IFNγ compared to those of the untreated control. Overall, the median cleavage probability was higher for the c20S than for the i20S (0.685 for the c20S and 0.574 for the i20S at the peptides’ C-terminus; 0.572 for the c20S and 0.432 for the i20S at the peptides’ N-terminus). Despite the large peptide overlap of immune epitopes, IFNγ significantly enhanced the C-terminal median cleavage probability of peptides derived from both the c20S (Figure 2A) and the i20S (Figure 2C), indicating an enhanced production of immune epitopes by both types of proteasomes. In contrast, IFNγ had no influence on the proteasomal median N-terminal cleavage probability of the c20S (Figure 2B) or of the i20S (Figure 2D).

**Figure 3:**
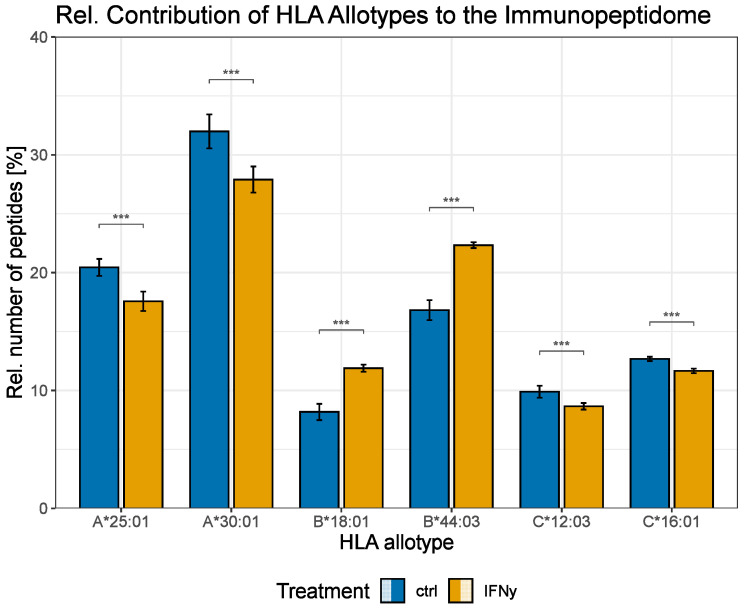
IFNγ affects relative contribution of the different HLA allotypes to the immunopeptidome. A549 cells were treated with IFNγ (yellow). Untreated cells served as a ctrl (blue). Grouped bar plot showing the relative number of peptides presented by the corresponding HLA allotypes ± SD based on six samples. Non-parametric aligned rank transform ANOVA was performed to test for statistical significance, followed by aligned ranked transform contrasts post hoc test and adjustment for multiple testing according to Benjamini–Hochberg, *** *p* < 0.001.

Since an increase in the C-terminal proteasomal cleavage probability of the c20S was not expected for the IFNγ-treated A549 cells, we further characterized the identified peptides, grouping them by their HLA allotype specificity (Figure 3). IFNγ significantly shifted the relative peptide contribution of the different HLA allotypes to the MHC class I peptide presentation. While, in relative terms, less peptides were derived from the HLA allotypes A*25:01, A*30:01, C*12:03 and C*16:01, the HLA allotypes B*18:01 and B*44:03 provided more peptides to the immunopeptidome after IFNγ stimulation. Although the IFNγ treatment of the A549 cells resulted in significant variations in the proportional peptide contribution of all HLA class I allotypes, only the HLA allotypes B*18:01, B*44:03 and C*16:01 exhibited a significant increase in the absolute number of presented peptides (Supplementary Figure S2).

**Figure 4:**
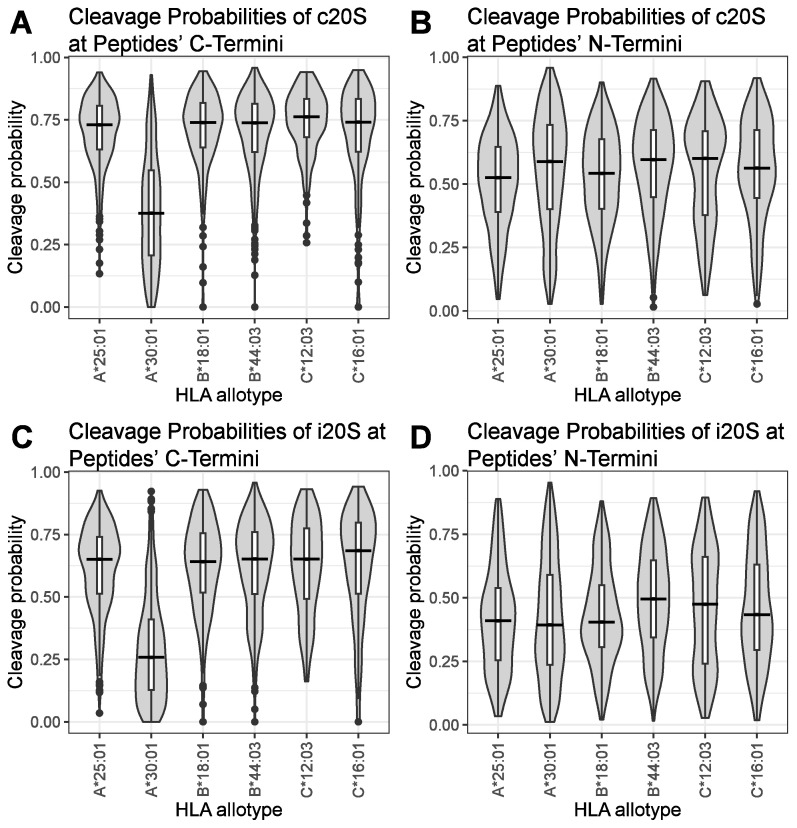
Reduced proteasomal cleavage probability at the C-terminus of peptides presented by HLA-A*30:01. Frequently identified MHC class I ligands from A549 cells treated with 75 IU/mL IFNγ and untreated cells were combined. The identified MHC class I peptides were grouped by their corresponding HLA allotype. Proteasomal cleavage probabilities at the C-terminus for c20S (**A**) or i20S (**C**) and at the N-terminus for c20S (**B**) or i20S (**D**) are depicted as trimmed violin plots containing a boxplot with median, first and third quartiles, and whiskers indicating data within the ±1.5 times interquartile range.

Next, we investigated whether the proteasomal cleavage probability of HLA ligands derived from A549 cells plus/minus treatment with IFNγ differs across HLA class I allotypes. Depicted are the C-terminal c20S (Figure 4A), N-terminal c20S (Figure 4B), C-terminal i20S (Figure 4C) and N-terminal i20S (Figure 4D) cleavage probabilities of the frequently identified immune epitopes presented by A549 cells grouped by their presenting HLA allotypes. Interestingly, peptides specific to the HLA allotype A*30:01 had significantly lower proteasomal cleavage probabilities for both the c20S (Figure 4A) and the i20S (Figure 4C) at the peptides’ C-terminus. The median cleavage probabilities for peptides derived from HLA-A*30:01 were 0.383 (c20S) and 0.253 (i20S). Peptides derived from the remaining HLA allotypes of the A549 cells had median C-terminal proteasomal cleavage probabilities ranging from 0.724 to 0.766 for the c20S and from 0.634 to 0.693 for the i20S. In contrast, the N-terminal c20S cleavage probability calculated for the HLA allotype A*30:01 was 0.591 and lies within the range of 0.516 to 0.603 calculated for the other HLA allotypes of the A549 cells (Figure 4B). Similarly, the median i20S N-terminal cleavage probability for peptides derived from HLA allotype A*30:01 was 0.397, close to the median i20S cleavage probabilities calculated for the other HLA allotypes (0.406–0.488) (Figure 4D). Thus, the proteasomal cleavage probability at the N-terminus of the MHC class I ligands derived from HLA-A*30:01 was not reduced.

HLA-A*30:01 presented immune epitopes with a reduced C-terminal proteasomal cleavage probability independently of the type of proteasome when compared to the other HLA allotypes expressed by the A549 cells. To evaluate whether this is specific to A549 cells or typical for certain HLA allotypes, we collected all MHC class I immune epitopes published in the IEDB [38]. In total, 554,617 HLA ligands were retrieved from the IEDB (Supplementary Table S3). Peptides with less than 8 amino acids, with more than 14 amino acids, with incomplete sequence information or containing a selenocystein were excluded from the binding score prediction for compatibility with the software MixMHCpred. The binding ranks for the remaining 497,254 peptides were calculated for all HLA allotypes supported by MixMHCpred, and only peptides with binding ranks smaller than or equal to 2% passed our peptide selection. The peptides were assigned to the HLA allotypes with their lowest binding rank. Furthermore, peptides were discarded if the corresponding HLA allotype had less than 200 peptides assigned to it or if the peptide was a duplicate. Additionally, we discarded peptides with different amino acid sequences or accessions in the SwissProt human database compared to the IEDB peptide list. The proteasomal cleavage probabilities for the c20S and the i20S were calculated for the entire SwissProt human database, and the cleavage scores were transferred to the IEDB peptide list. This filtering eventually resulted in 88,513 HLA ligands assigned to 71 HLA allotypes with the corresponding N- and C-terminal proteasomal cleavage probabilities for the c20S and the i20S (Supplementary Table S4).

**Figure 5:**
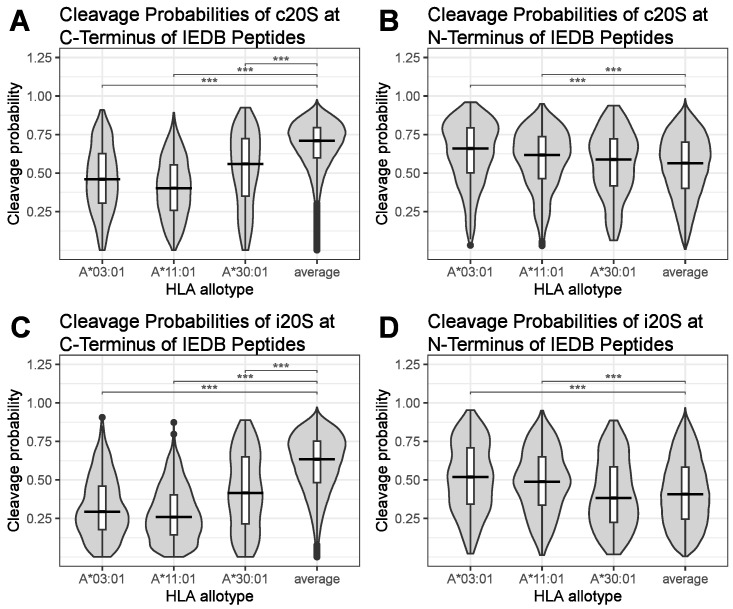
HLA allotypes with lowest C-terminal proteasomal cleavage probabilities derived from the IEDB. Peptides were collected from the IEDB and grouped by their corresponding HLA allotypes. C- and N-terminal cleavage probabilities of the peptides for processing by the c20S or i20S were calculated using the software Pepsickle. Depicted are the three HLA allotypes with the lowest C-terminal cleavage probability for the c20S (**A**) and their corresponding N-terminal c20S cleavage probabilities (**B**). Depicted are the three HLA allotypes with the lowest C-terminal cleavage probability for the i20S (**C**) and the corresponding N-terminal cleavage probability for the i20S (**D**). Average indicates the corresponding distribution of all analyzed peptides and serves as a reference. Trimmed violin plots containing a boxplot with median, first and third quartiles, and whiskers indicating data within the ±1.5 times interquartile range. Non-parametric Mood’s median test followed by pairwise median test and adjustment for multiple testing was performed to test for statistical significance, *** *p* < 0.001.

Next, we compared the different HLA-specific median C- and N-terminal c20S and i20S peptide cleavage probabilities to the corresponding median cleavage probabilities of all peptides (average). Notably, many HLA allotypes presented MHC class I ligands with significantly different C-terminal c20S (Supplementary Figures S3A and S5A), N-terminal c20S (Supplementary Figures S3B and S5B), C-terminal i20S (Supplementary Figures S3C and S5C) and N-terminal i20S (Supplementary Figures S3D and S5D) median proteasomal cleavage probabilities. However, the effect size was most significant for the C-terminal median cleavage probabilities of the three HLA allotypes A*03:01, A*11:01 and A*30:01 (Figure 5A,C). This is the case independently of the type of proteasome. In contrast, the median proteasomal cleavage probabilities for the N-termini of these HLA ligands were not decreased compared to the average, but they were significantly increased in the case of peptides presented by HLA-A*03:01 and HLA-A*11:01 (Figure 5B,D).

The analysis of the peptides derived from the IEDB confirmed our initial observation obtained with the ligands of HLA-A*30:01 derived from the A549 cells. Furthermore, we identified two more HLA allotypes with decreased C-terminal proteasomal cleavage probabilities, namely, A*03:01 and A*11:01. The three HLA allotypes with the lowest C-terminal immune epitope cleavage probabilities are the allotypes of the HLA-A gene. However, it should be noted that HLA-B allotypes like HLA-B*45:01 or HLA-B*55:01 also exhibited a noticeably diminished C-terminal immune epitope cleavage probability (Supplementary Figure S3A,C). The immune epitopes presented by HLA-B*27 (HLA-B*27:01; HLA-B*27:02; HLA-B*27:04; HLA-B*27:05; HLA-B*27:06; HLA-B*27:07), HLA-C*06:02 and HLA-C*07:02 had a prominently reduced cleavage probability at the peptides’ N-terminus, and this was exclusive to the cleavage probability of the i20S (Supplementary Figure S3D).

**Figure 6:**
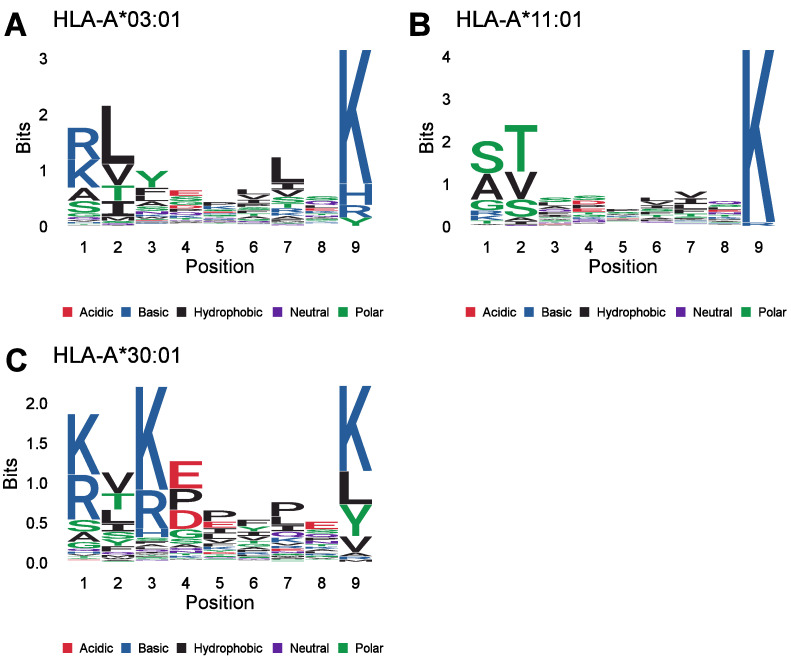
Motif comparison of HLA allotypes presenting peptides with low C-terminal proteasomal cleavage probabilities. Peptides derived from the IEDB presented by the HLA allotypes HLA-A*03:01 (**A**), HLA-A*11:01 (**B**) and HLA-A*30:01 (**C**) were aligned using Gibbs clustering [36]. Depicted are sequence logos of the corresponding peptide alignments.

To further characterize the ligands of these HLA allotypes, we analyzed the corresponding peptide motifs of the peptides derived from the IEDB. The peptide motifs specific to the HLA allotypes A*03:01 (Figure 6A), A*11:01 (Figure 6B) and A*30:01 (Figure 6C) have a common C-terminal lysine (K). These peptide motifs also resemble the peptide motifs published in the MHC Motif Atlas. Most importantly, the MHC Motif Atlas confirms the C-terminal K in the peptide motif of these MHC class I ligands [43]. A C-terminal K is also part of the binding motifs of the HLA allotypes HLA-A*03:02, HLA-A*11:02 and HLA-A*34:02, as described in the MHC Motif Atlas [43]. However, none of the peptides derived from the IEDB were identified as ligands of one of these HLA allotypes. The HLA class I allotype HLA-A*68:01 is reported to have a C-terminal K or a C-terminal arginine (R) as part of its binding motif, and 1060 peptides derived from the IEDB were sorted into HLA-A*68:01 [43]. However, only 65 of these MHC class I ligands have a C-terminal K, whereas 992 amino acids have a C-terminal arginine residue, and 3 peptides have a C-terminal leucine residue. Accordingly, the C-terminal proteasomal cleavage score for the ligands of HLA-A*68:01 was not markedly reduced compared to that of the other MHC class I ligands (Supplementary Figure S3A,C). Furthermore, we created peptide motifs covering two additional amino acids C-terminally for these HLA allotypes to investigate whether there are common features in the two amino acid positions following the cleavage sites of the peptides (Supplementary Figure S4A–C). However, no specific amino acid was enriched C-terminally of the binding motifs of these HLA allotypes. In summary, we identified three HLA allotypes with affinities for peptides with a reduced proteasomal cleavage probability at their C-terminus, and these peptides have a C-terminal K in common.

## 4. Discussion

The analysis of the C-terminal cleavage probabilities of peptides derived from either A549 cells or the IEDB revealed reduced C-terminal cleavage probabilities for certain HLA allotypes, namely, HLA-A*03:01, HLA-A*11:01 and HLA-A*30:01. Furthermore, the reduced cleavage probability correlated with the presence of a C-terminal K in the binding motifs of these HLA allotypes. 

Previously, the calculation of the proteasomal cleavage probability has been successfully used to predict MHC class I ligands, i.e., those derived from SARS-CoV-2 [44]. In contrast, we calculated the proteasomal cleavage probabilities of the immune epitopes presented by the lung cancer cell line A549 cells plus/minus treatment with IFNγ. IFNγ induces the expression of the i20S and shapes the antigen presentation by MHC class I [14,17]. We therefore attempted to estimate i20S activity by comparing the proteasomal cleavage probability of IFNγ-treated A549 cells to an untreated control using Pepsickle, software for the prediction of proteasomal cleavage probability [29].

The Pepsickle software tool is based on an ensemble deep-learning algorithm and provides two different modes built by different types of training data [29]. The in vivo model is based on immune epitopes derived from three databases, namely, the IEDB [38], AntiJen [45] and SYFPEITHI [46], as well as data published as part of a research article [47]. In contrast, the in vitro model is based on in vitro 20S degradation experiments [29]. The performance of both models was tested previously by predicting degradation products and comparing them to immune epitopes published by the group of Michal Bassani-Sternberg [48]. While the in vivo model performs better than the in vitro model in predicting the proteasomal cleavage probability at the C-terminus of MHC class I ligands, it makes the assumption that all immune epitopes are of proteasomal origin. Furthermore, the N-terminal cleavage prediction is less accurate since immune epitopes are often N-terminally extended and need further processing by other proteases and aminopeptidases during peptide loading [25,29]. Thus, we decided to perform our analysis with the in vitro model, which is more accurate in predicting the proteasomal cleavage probability at the N-terminus and is less biased about the origin of immune epitopes.

The MHC class I peptides from the IEDB had to be filtered to allow for the grouping of the peptides to their antigen-presenting class I HLA allotype and to enable the matching of the peptides’ sequences and the protein sequences of the SwissProt human database (which was used for the proteasomal cleavage probability prediction). The filtering of the immune epitopes derived from the IEDB reduced the peptides from 554,617 HLA ligands to 88,513 HLA ligands. The largest number of immune epitopes was excluded since they did not match the binding requirements as calculated with the MixMHCpred software [33,34]. To ensure the integrity and validity of our results, the filtering process was limited to technical considerations, avoiding any further manipulation or distortion of the data. Consequently, 8- to 14mer peptides passed filtering, although MHC class I peptides are typically 8 to 11 amino acids long [34]. However, the length of peptides is factored into the affinity prediction of the software MixMHCpred [33,34]. MixMHCpred calculates the binding scores of peptides to different HLA allotypes and uses these binding scores to assess the binding rank. The binding rank is the likelihood that a random set of peptides would result in a similarly high binding score [33,34]. While MixMHCpred supports the most frequent HLA allotypes, less frequent HLA allotypes are not supported, resulting in the rejection of a large proportion of immune epitopes derived from the IEDB. However, we were able to assign MHC class I ligands to 71 HLA class I allotypes with more than 200 peptides per allotype. The description of additional peptide binding motifs and implementation into the corresponding software tools for binding score prediction will help to calculate even more HLA allotype-specific proteasomal cleavage scores in the future.

Although the induction of the i20S by treatment with IFNγ led to an increase in the cleavage probability of the i20S at the C-terminus of the immune epitopes, this cannot be attributed to an enhanced activity of the i20S since the C-terminal cleavage probability for the c20S was also increased. Besides the induction of the i20S, IFNγ also affected the relative contribution of the different HLA allotypes and genes to the immunopeptidome. Most markedly, it reduced the relative antigen presentation of HLA-A and enhanced antigen presentation by HLA-B. It is worth noting that the shift in the relative contribution of HLA allotypes to the immunopeptidome mostly originates from an enhanced peptide contribution of HLA-B allotypes, while the absolute contribution of HLA-A to the immunopeptidome is not significantly altered. This reproduces the findings of Aaron Javitt and colleagues, who demonstrated that IFNγ induces the expression of HLA-B in A549 cells [17]. The median proteasomal cleavage probability inherently depends on the relative peptide composition. Since HLA-A*30:01 presented immune epitopes with a noticeably reduced C-terminal proteasomal cleavage probability for both types of proteasomes, the c20S and the i20S, the increased proteasomal cleavage probabilities most likely resulted from the relative shifts of the HLA contribution to the immunopeptidome. Thus, we cannot comment on the proteasomal cleavage activity following the IFNγ treatment of the A549 cells by comparing the C-terminal c20S or i20S median cleavage probabilities of their MHC class I ligands. 

The C-terminal proteasomal cleavage probability of 243 immune epitopes suggested processing exclusive to the c20S, while the C-terminal cleavage probability of 8 immune epitopes suggested processing exclusive to the i20S. Previously, in vitro digestion experiments with c20S and i20S indicated quantitative rather than qualitative differences in processing by the two different types of proteasomes, and 957 MHC class I ligands have a C-terminus with a cleavage probability, proposing processing by both the c20S and the i20S [49]. However, it has been shown that both types of proteasomes can differ in their substrate processing, resulting in distinct MHC class I ligands depending on the type of proteasome [8]. Furthermore, a murine subset of CD8^+^ T cells targeting an MHC class I ligand derived from the GP protein of the lymphocytic choriomeningitis virus requires processing by the i20S, indicating that a unique set of MHC class I ligands is produced by this type of proteasome [50]. Future experiments should clarify whether the identified immune epitopes are exclusively produced by one type of proteasome, or whether the observed differences in the cleavage probability result in quantitative differences of the generated peptides. 

The overall composition of the peptides presented by the IFNγ-treated A549 cells differed noticeably from that of the peptides presented by the untreated control. IFNγ treatment affects the expression of many components of the antigen presentation machinery, like the expression of TAP or the level of MHC class I itself [17,51]. Furthermore, it modifies the cellular protein expression in general, which is also reflected in the immune epitopes presented by the cells [51]. While IFNγ treatment induces a plethora of pro-inflammatory proteins, the majority of proteins are assumed to be non-differentially expressed [52]. This is also true for A549 cells, where most proteins are not differentially expressed, as shown here by the proteome analysis. Consequently, IFNγ should exert its influence on antigen presentation by modulating the antigen processing and loading machinery rather than the expression of source proteins.

While the C-terminal cleavage probabilities of the c20S and the i20S overall showed similar tendencies, some HLA allotypes, like HLA-B*27, HLA-C*06:02 or HLA-C*07:02, had pronounced differences in their N-terminal cleavage probabilities for the c20S and the i20S. HLA-B*27 is described as a risk factor for several autoimmune diseases, like ankylosing spondylitis [53], reactive arthritis [54] and uveitis [55] (for a summary, see [56]). Most MHC class I ligands are N-terminally extended and require further processing during peptide loading by other proteases or aminopeptidases, respectively [25,26]. The reduced cleavage probability of the i20S at the N-terminus of the immune epitopes presented by these HLA allotypes could further increase the likelihood of N-terminally extended MHC class I ligands. The binding motifs of these HLA allotypes have in common an arginine at position two of their MHC class I peptide motif [33]. It has been shown previously that both types of proteasomes, the c20S and the i20S, can differ in their substrate processing [8]. The software Pepsickle considers the eight amino acid contexts at each cleavage site spanning almost the whole length of the MHC class I immune epitopes [29]. One possible explanation for the low N-terminal cleavage probability of the immune epitopes presented by HLA-B*27 could be that the peptide motifs of these HLA allotypes could promote the destruction of the immune epitope by the i20S. Thus, only peptides that are not cleaved within the immune epitope itself could be presented, resulting in N-terminally extended peptides. Additionally, the allotypes HLA-B*27 and HLA-C*06:02 are reported to present a subset of MHC class I ligands longer than nonamers [57,58]. Thus, N-terminally extended peptides originating from i20S protein degradation could serve as substrates for these two HLA allotypes. In contrast, the HLA allotype HLA-C*07:02 presents peptides with the typical length distribution of MHC class I [59]. Further investigation is needed to elaborate on the low N-terminal cleavage probability exclusive to the i20S for the immune epitopes presented by HLA-B*27, HLA-C*06:02 or HLA-C*07:02.

Our analysis suggested that the HLA allotypes HLA-A*03:01, HLA-A*11:01 and HLA-A*30:01, sharing a C-terminal K, have a reduced C-terminal proteasomal processing probability. K is a basic amino acid containing an ε-amino group. Furthermore, it is the second most post-translationally modified amino acid after serine. One well-known modification of K is K48-linked polyubiquitination, which targets a protein for proteasomal degradation (for a summary, see [60]). Recently, researchers have been investigating the influence of various post-translational modifications on protein turnover [61]. However, to the best of our knowledge, no study has addressed the influence of post-translational modifications on the proteasomal cleavage pattern. Previously, it has been shown using a synthetic peptide library that processing by the proteasome of a substrate with a K at the proteasomal cleavage position P1 is disadvantaged [62]. However, the association of the proteasome with the regulative subunit REGγ (a homoheptamer formed by PSME3 proteins) specifically activates the trypsin-like proteasomal subunit promoting cleavage after large basic amino acids like K. Processing with a K at the proteasomal cleavage position P1 leaves the K at the C-terminus of the cleavage site [62]. PSME3 is expressed in the nucleus, where it is involved in the proteasomal degradation of peptides derived from non-conventional translation products (the translation of introns, intron/exon junctions, and 5′ and 3′ untranslated regions) [63]. A549 cells express low levels of PSME3 [63]. While PSME3 should promote the generation of peptides suitable for presentation by HLA-A*30:01, the exogenous overexpression of PSME3 was found to impair the antigen presentation of A549 cells by inducing the proteasomal destruction of peptides [63]. Thus, future experiments should clarify whether HLA-A*30:01 ligands are created by proteasomal trypsin-like activity or whether HLA-A*30:01 prefers peptides that are generated either independently of the proteasome or by proteases that further trim proteasomal degradation products. While REGγ specifically activates trypsin-like proteasomal activity, the cytosolic heteroheptameric PA28αβ regulator, formed by the genes *PSME1* and *PSME2*, activates all three catalytically active subunits of the proteasome [64]. Furthermore, IFNγ has been shown to enhance proteasomal activity [65]. However, differences in proteasomal activity cannot explain the reduced cleavage probability of both the c20S and the i20S for the ligands presented by HLA-A*30:01.

So far, it remains unclear to which extent the diminished C-terminal cleavage probability of both types of proteasomes at the C-terminus of the peptides presented by HLA-A*03:01, HLA-A*11:01 or HLA-A*30:01 impedes or impairs the proteasomal production of these peptides. However, the markedly reduced C-terminal cleavage probabilities of the peptides presented by the HLA allotypes HLA-A*03:01, HLA-A*11:01 and HLA-A*30:01 could suggest that these HLA allotypes favor immune epitopes of proteasome-independent origin. The proteasome-independent HIV-1 immune epitope Nef(73-82), processed by tripeptidyl peptidase II, is presented by both HLA-A*03:01 and HLA-A*11:01 [12]. 

The analysis of the immunopeptidome of the A549 cells allowed us to discern a low proteasomal processing probability of MHC class I ligands presented by HLA-A*30:01. The immune epitopes derived from the IEDB further revealed that a C-terminal K correlates with a reduced proteasomal processing probability. The finding presented herein heavily depends on a comparison of two biostatistical algorithms that both rely on amino acid sequences of MHC class I ligands. Although these algorithms have proven powerful in either calculating peptide affinities to MHC class I or predicting proteasomal cleavage probabilities based on the amino acid context at the cleavage sites [29,33,34], we are aware that further experimental evidence is necessary to fully characterize the origin of MHC class I ligands with a C-terminal lysine residue. Previously, antigen loading by HLA-A*03 and HLA-A*11 has been shown to be less sensitive to treatment with the proteasome inhibitors z-LLL and lactacystin, indicating a proteasome-independent mode of action [66]. Immunopeptidomics requires substantial sample quantities. The inhibition of the proteasome is not well tolerated by cells and might interfere with the required sample quantity. Thus, different assays are necessary to investigate the contribution of the c20S and the i20S to the processing of ligands for presentation by HLA-A*03:01, HLA-A*11:01 and HLA-A*30:01. Future studies will decipher the origin of MHC class I ligands with a C-terminal K by analyzing the MHC class I loading of B-LCL cells homozygous for these HLA allotypes. The MHC class I peptide loading of these cells should be less sensitive to the inhibition of the proteasome compared to cells expressing other HLA allotypes.

## 5. Conclusions

In conclusion, this study provides insights into the proteasomal processing of immune epitopes, highlighting HLA class I allotype-specific differences. While the majority of MHC class I immune epitopes are proteasome-dependent, there might by HLA allotypes that favor non-canonical peptide sources.

## Figures and Tables

**Table 1:** Induction of i20S and components of the MHC class I antigen presentation machinery by treatment of A549 cells with IFNγ.

Gene Symbol	Description	Log_2_ Abundance Ratio: IFNΓ/Ctrl	Adj. *p*-Value: IFNΓ/CTRL
PSMB8	Proteasome subunit beta type-8	1.97	2.41 × 10^−6^
PSMB9	Proteasome subunit beta type-9	2.56	5.13 × 10^−11^
PSMB10	Proteasome subunit beta type-10	2.89	2.70 × 10^−12^
TAP1	Antigen peptide transporter 1	3.31	3.65 × 10^−16^
TAP2	Antigen peptide transporter 2	3.00	9.66 × 10^−14^
HLA-A	HLA class I histocompatibility antigen, A alpha chain	1.36	7.47 × 10^−3^
HLA-B	HLA class I histocompatibility antigen, B alpha chain	1.68	4.72 × 10^−6^
HLA-C	HLA class I histocompatibility antigen, C alpha chain	1.80	1.95 × 10^−5^
HLA-E	HLA class I histocompatibility antigen, alpha chain E	1.99	6.32 × 10^−6^
B2M	Beta-2-microglobulin	2.40	1.09 × 10^−9^
TAPBP	Tapasin	2.00	6.69 × 10^−6^
ERAP1	Endoplasmic reticulum aminopeptidase 1	1.16	4.63 × 10^−2^

## Data Availability

The data presented in this study are available in the Supporting Information. Mass spectrometry raw files are available in an online repository under https://www.ebi.ac.uk/pride/, accessed on 22 August 2023. The project accession is PXD044235.

## Supplementary Materials

The following supporting information can be downloaded at https://www.mdpi.com/article/10.3390/biom13091300/s1, Figure S1: Differential protein expression in A549 cells following treatment with IFNγ; Figure S2: IFNγ affects absolute contribution of HLA allotypes to the immunopeptidome; Figure S3: All C- and N-terminal proteasomal cleavage probabilities of immune epitopes derived from the IEDB; Figure S4: C-terminally extended motif comparison of HLA allotypes presenting peptides with low C-terminal proteasomal cleavage probabilities; Supplementary Table S1: List of peptides identified via immunopeptidomics; Supplementary Table S2: Proteome of A549 cells treated with and without IFNγ; Supplementary Table S3: MHC class I epitopes derived from the IEDB; Supplementary Table S4: N- and C-terminal proteasomal cleavage probabilities of IEDB peptides.
