# Supplementary material for: A Lysine Residue at the C-Terminus of MHC Class I Ligands Correlates with Low C-Terminal Proteasomal Cleavage Probability
Source: Biomolecules. 2023 Aug 24;13(9):1300. doi: 10.3390/biom13091300 (PMC10527444; doi:10.3390/biom13091300)
Supplement: Supplementary file 1 [file biomolecules-13-01300-s001.zip › Supplementary_Schmalen_et_al_revised.pdf]

biomolecules

MDPI

Supporting Information

Supplementary Figures

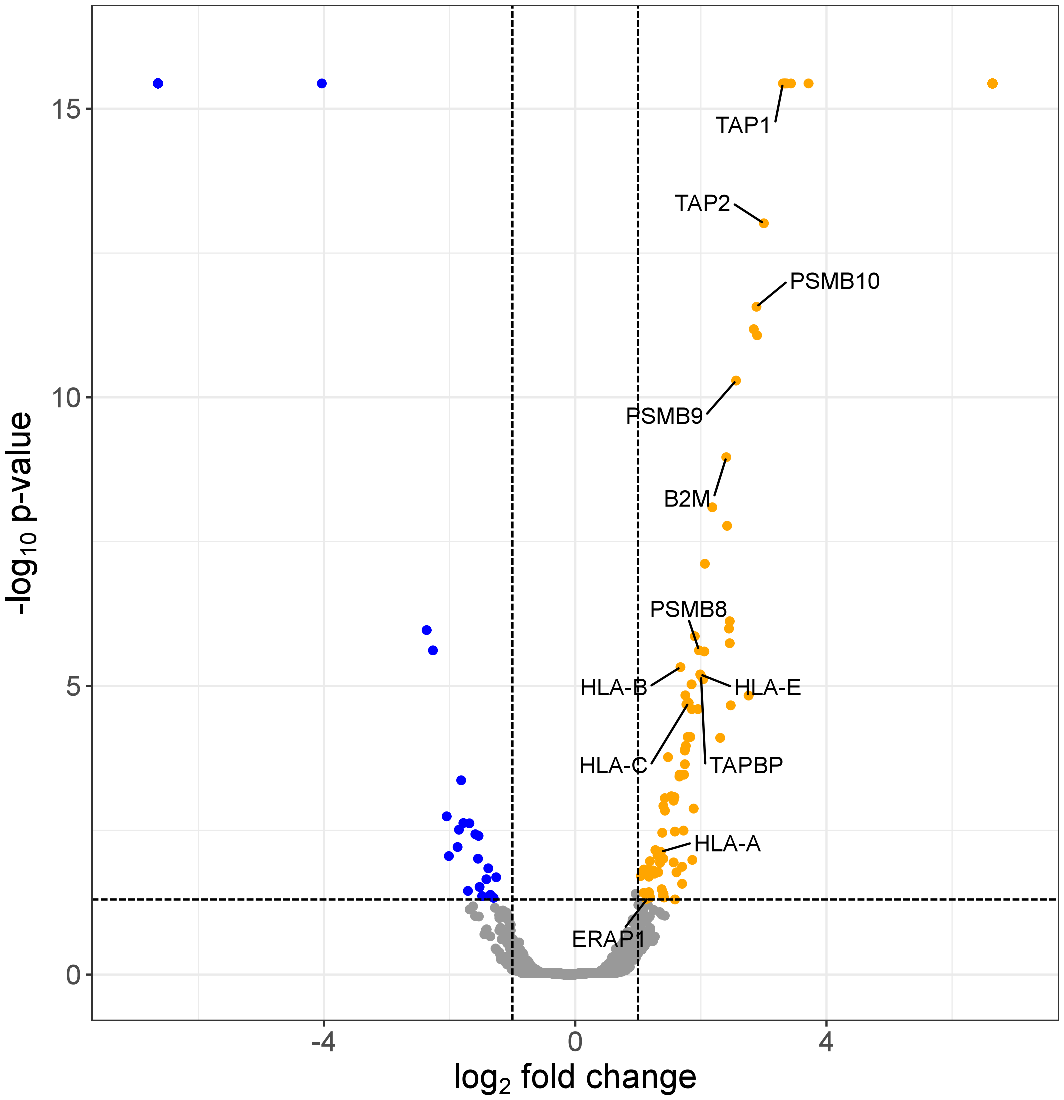

Supplementary Figure S1: Differential protein expression in A549 cells following treatment with IFNγ. Volcano plot of proteins identified in A549 cells after treatment with IFNγ. Significantly upregulated proteins are depicted in orange (log2 fold change ≥ 1; adjusted p-value ≤ 0.05). Significantly downregulated proteins are depicted in blue (log2 fold change ≤ -1; adjusted p-value ≤ 0.05). Differentially expressed proteins of the immunoproteasome and of components of the antigen presentation machinery are labeled with the corresponding gene symbol.

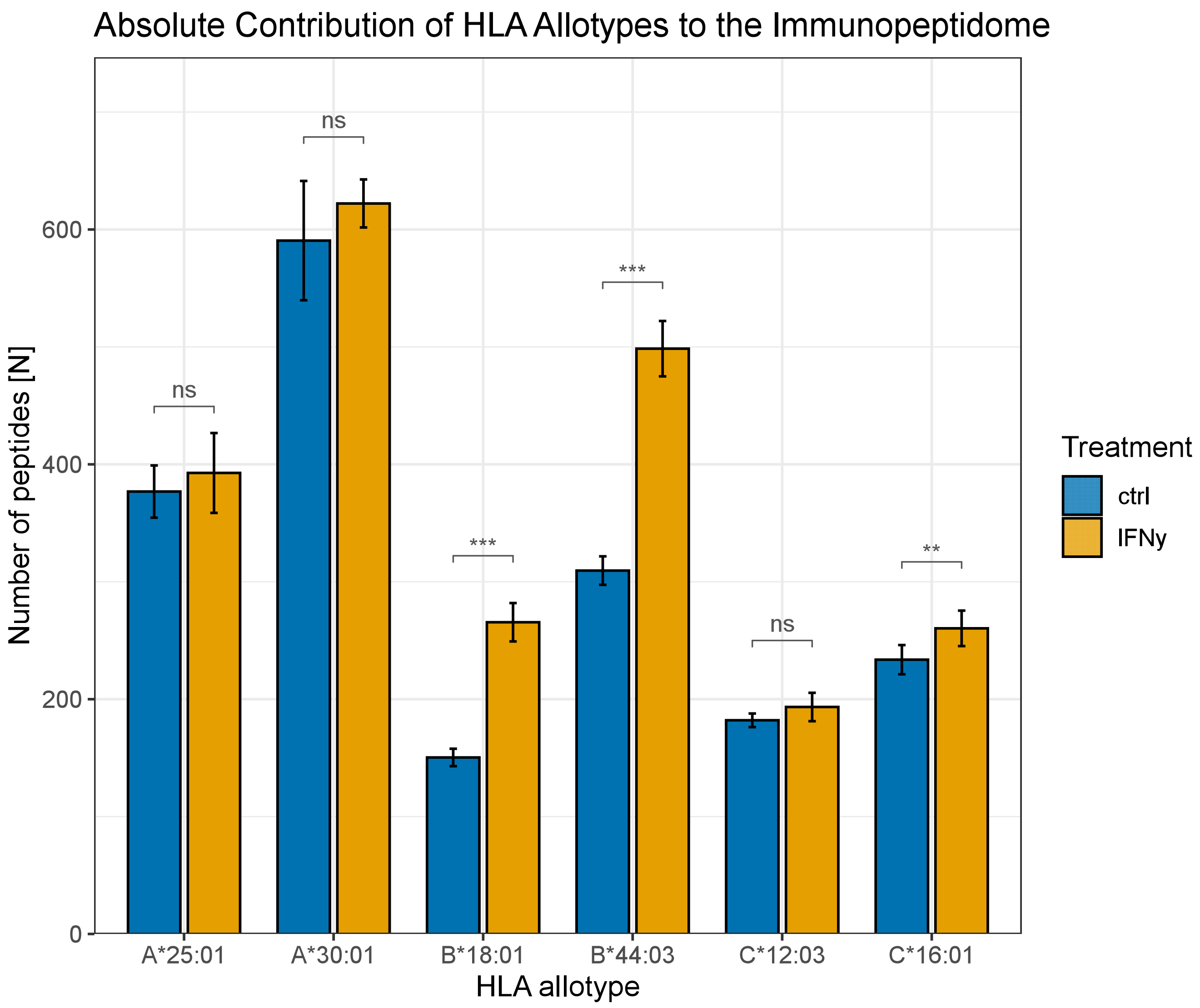

Supplementary Figure S2: IFNγ affects absolute contribution of HLA allotypes to the immunopeptidome. A549 cells were treated with IFNγ (orange). Untreated cells served as a ctrl (blue). Grouped bar plot showing the average number of peptides presented by the corresponding HLA allotypes ± SD based on six samples. Non-parametric aligned rank transform anova was performed to test for statistical significance, followed by aligned ranked transform contrasts post hoc test and adjustment for multiple testing, ***$p<0.001$, **$p<0.01$, ns $p\geq0.05$.

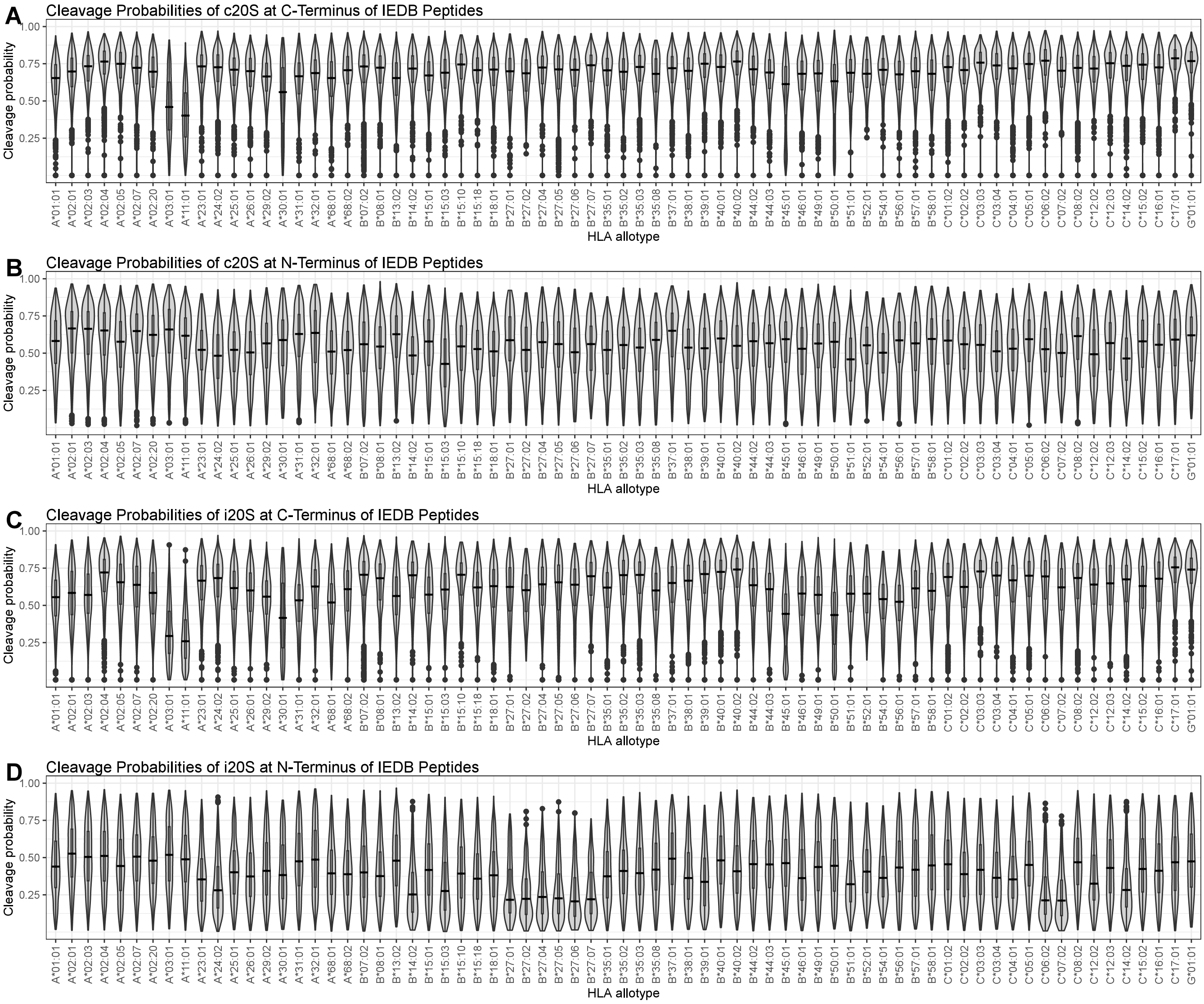

Supplementary Figure S3. All C- and N-terminal proteasomal cleavage probabilities of immune epitopes derived from the IEDB. Immune epitopes derived from the IEDB were clustered corresponding to their HLA allotype with the lowest MHC class I binding rank. Cleavage probabilities for the c20S were calculated for the C-terminus (**A**) and N-terminus (**B**) of these immune epitopes. Furthermore, cleavage probabilities for the i20S were calculated for the C-terminus (**C**) and N-terminus (**D**) of these immune epitopes. Depicted are trimmed violin plots containing a boxplot with median, first and third quartile and whiskers indicating data within the ± 1.5 times interquartile range.

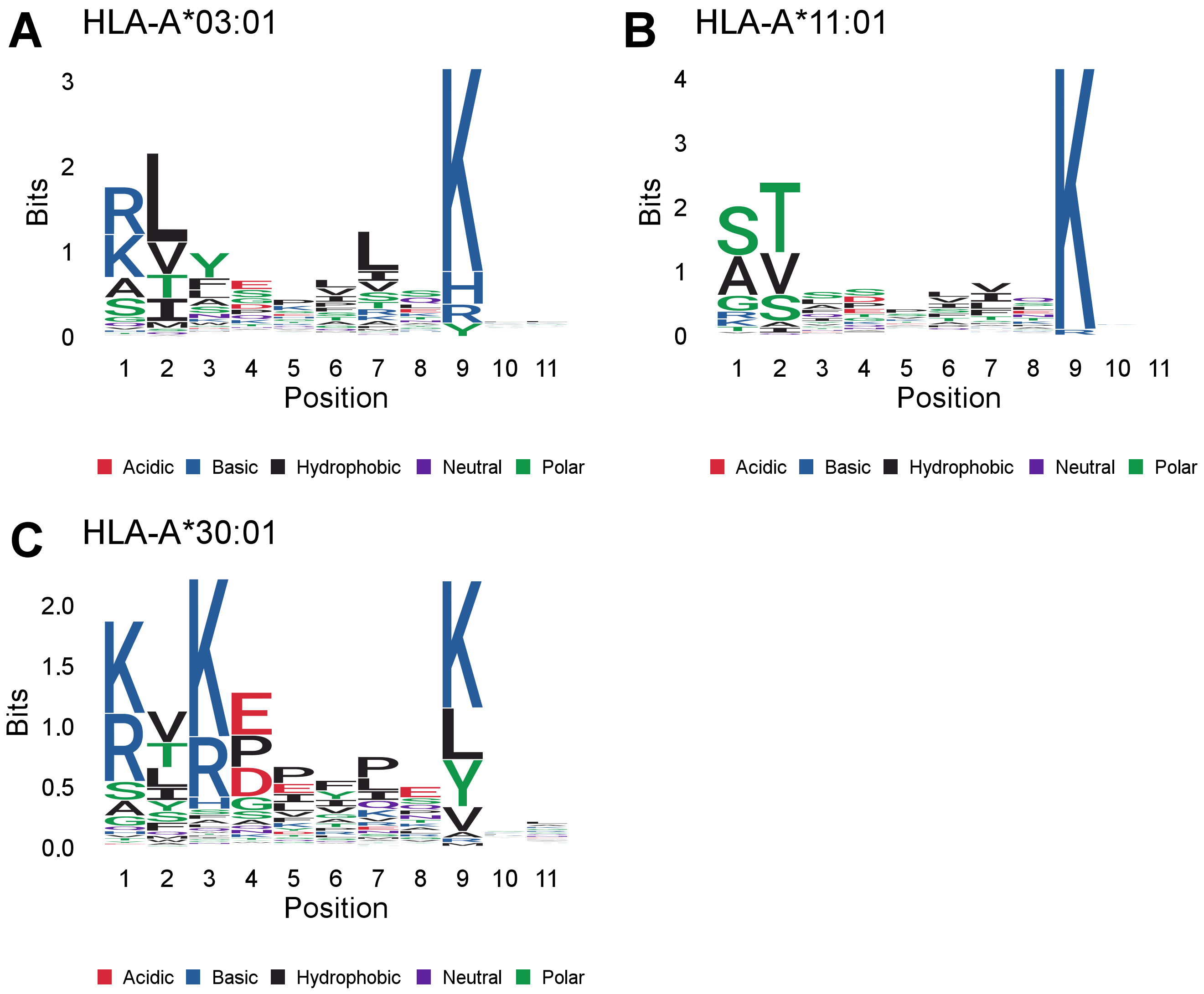

Supplementary Figure S4. C-terminally extended motif comparison of HLA allotypes presenting peptides with low C-terminal proteasomal cleavage probability. Peptides derived from the IEDB presented by the HLA allotypes HLA-A*03:01 (**A**), HLA-A*11:01 (**B**), and HLA-A*30:01 (**C**) were aligned by Gibbs clustering [1]. Depicted are C-terminally extended sequence logos of the corresponding peptide alignments.

Reference:

1. Andreatta, M.; Alvarez, B.; Nielsen, M. GibbsCluster: unsupervised clustering and alignment of peptide sequences. *Nucleic Acids Res* **2017**, *45*, W458-W463, doi:10.1093/nar/gkx248.

Disclaimer/Publisher's Note: The statements, opinions and data contained in all publications are solely those of the individual author(s) and contributor(s) and not of MDPI and/or the editor(s). MDPI and/or the editor(s) disclaim responsibility for any injury to people or property resulting from any ideas, methods, instructions or products referred to in the content.
